# Automatic differentiation of ruptured and unruptured intracranial aneurysms on computed tomography angiography based on deep learning and radiomics

**DOI:** 10.1186/s13244-023-01423-8

**Published:** 2023-05-04

**Authors:** Junbang Feng, Rong Zeng, Yayuan Geng, Qiang Chen, Qingqing Zheng, Fei Yu, Tie Deng, Lei Lv, Chang Li, Bo Xue, Chuanming Li

**Affiliations:** 1 Medical Imaging Department, Chongqing University Central Hospital, No. 1, Jiankang Road, Yuzhong District, Chongqing, 400014 China; 2 Medical Imaging Department, Chongqing Emergency Medical Center, No. 1, Jiankang Road, Yuzhong District, Chongqing, 400014 China; 3 Department of Radiology, The Second Affiliated Hospital of Chongqing Medical University, No. 74 Linjiang Road, Yuzhong District, Chongqing, 400010 China; 4 Department of Research and Development, Shukun (Beijing) Network Technology Co., Ltd, No. Room 801, Jinhui Building, Qiyang Road, Chaoyang District, Beijing, 200232 China

**Keywords:** Computed tomography angiography, Intracranial aneurysm, Rupture, Deep learning, Radiomics

## Abstract

**Objectives:**

Rupture of intracranial aneurysm is very dangerous, often leading to death and disability. In this study, deep learning and radiomics techniques were used to automatically detect and differentiate ruptured and unruptured intracranial aneurysms.

**Materials and methods:**

363 ruptured aneurysms and 535 unruptured aneurysms from Hospital 1 were included in the training set. 63 ruptured aneurysms and 190 unruptured aneurysms from Hospital 2 were used for independent external testing. Aneurysm detection, segmentation and morphological features extraction were automatically performed with a 3-dimensional convolutional neural network (CNN). Radiomic features were additionally computed via pyradiomics package. After dimensionality reduction, three classification models including support vector machines (SVM), random forests (RF), and multi-layer perceptron (MLP) were established and evaluated via area under the curve (AUC) of receiver operating characteristics. Delong tests were used for the comparison of different models.

**Results:**

The 3-dimensional CNN automatically detected, segmented aneurysms and calculated 21 morphological features for each aneurysm. The pyradiomics provided 14 radiomics features. After dimensionality reduction, 13 features were found associated with aneurysm rupture. The AUCs of SVM, RF and MLP on the training dataset and external testing dataset were 0.86, 0.85, 0.90 and 0.85, 0.88, 0.86, respectively, for the discrimination of ruptured and unruptured intracranial aneurysms. Delong tests showed that there was no significant difference among the three models.

**Conclusions:**

In this study, three classification models were established to distinguish ruptured and unruptured aneurysms accurately. The aneurysms segmentation and morphological measurements were performed automatically, which greatly improved the clinical efficiency.

**Clinical relevance statement:**

Our fully automatic models could rapidly process the CTA data and evaluate the status of aneurysms in one minute.

**Graphical Abstract:**

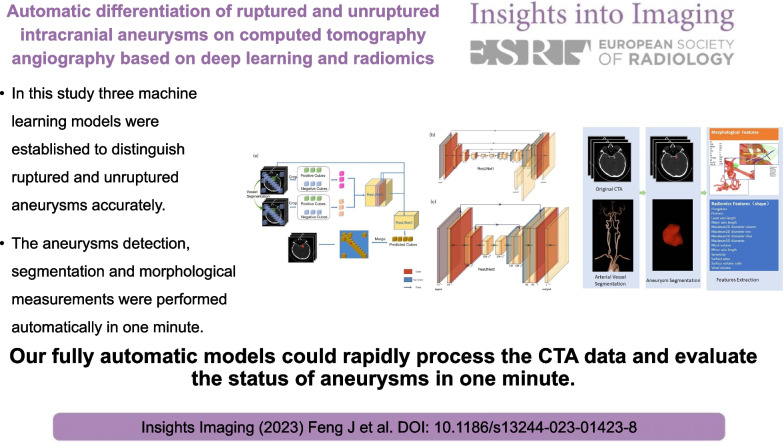

## Key points

1. Three machine learning models were established to distinguish ruptured and unruptured aneurysms.
2. The aneurysms detection, segmentation and morphological measurements were performed automatically.
3. Thirteen morphological and radiomics features were proved associated with aneurysm rupture.

## Introduction

Intracranial aneurysm (IA) is a common and life-threatening serious neurological disease caused by abnormal swelling or dilation of the walls of intracranial arteries [1]. With the rapid development of medical imaging technology, more and more IAs have been found, and the incidence rate among general population is more than 3%. Aneurysms rupture can lead to serious consequences, with a mortality rate of 40%. Survivors may suffer from long-term neurological sequelae and life quality decline [2]. In clinical practice, about 85% of spontaneous subarachnoid hemorrhage (SAH) is caused by intracranial aneurysm rupture [3]. However, some patients with ruptured aneurysms did not show subarachnoid hemorrhage. On the other hand, for patients with multiple aneurysms, although there is subarachnoid hemorrhage, it is hard to determine which aneurysm ruptures. So, accurate identification of IA status is essential for clinical treatment and prognosis evaluation [4, 5]. Ruptured aneurysms demand prompt surgery while the treatment of unruptured aneurysms is controversial. Some unruptured aneurysms may remain asymptomatic for life [6], and either intravascular or microsurgical treatment may add risks of complications [7].

Computed tomography angiography (CTA) is a non-invasive, efficient and accurate method for IA detection [8]. Although digital subtraction angiography (DSA) remains the gold standard, its invasion and time-consuming limited the clinical application, besides the high cost and complications [9]. At present, the accuracy of CTA in detecting IA has reached 97%–100% [10] and is of great potential to replace DSA [11]. Some studies [12, 13] have focused on the morphological difference between ruptured and unruptured aneurysms in CTA and found that several morphological features were associated with aneurysm rupture, such as neck diameter, aneurysm length, height, width, surface area, volume, etc. Other studies have proved radiomics valuable in classifying ruptured and unruptured intracranial aneurysms with CTA images [14]. However, the accuracies of these studies were not high enough, lacking large samples and external independent verification. On the other hand, both aneurysm segmentation and morphological measurement needed manual operation, which was very time-consuming and highly depended on the experience of radiologists. It might take about 30 min to complete the reconstruction, segmentation and analysis of each case.

Convolutional neural network (CNN) [15] is one of the most commonly used deep learning methods. It has shown great potential in medical fields,such as disease detection, segmentation, diagnosis and prediction [16, 17]. In previous research, we had developed an artificial intelligence model based on CNN to identify the arteries in head and detect intracranial aneurysms [15, 16]. The whole automatic segmentation and reconstruction process took less than a minute. In this study, large and multi-center samples were collected. The detection, segmentation and morphological parameters measurement of intracranial aneurysms were automatically performed and different machine learning models were established and independently tested in external dataset to help the differentiation of ruptured and unruptured intracranial aneurysms.

## Materials and methods

### Ethical statement

This study was approved by the Research Ethics Committee of Chongqing University Central Hospital, and informed consent was waived.

### Patients

**Fig. 1:**
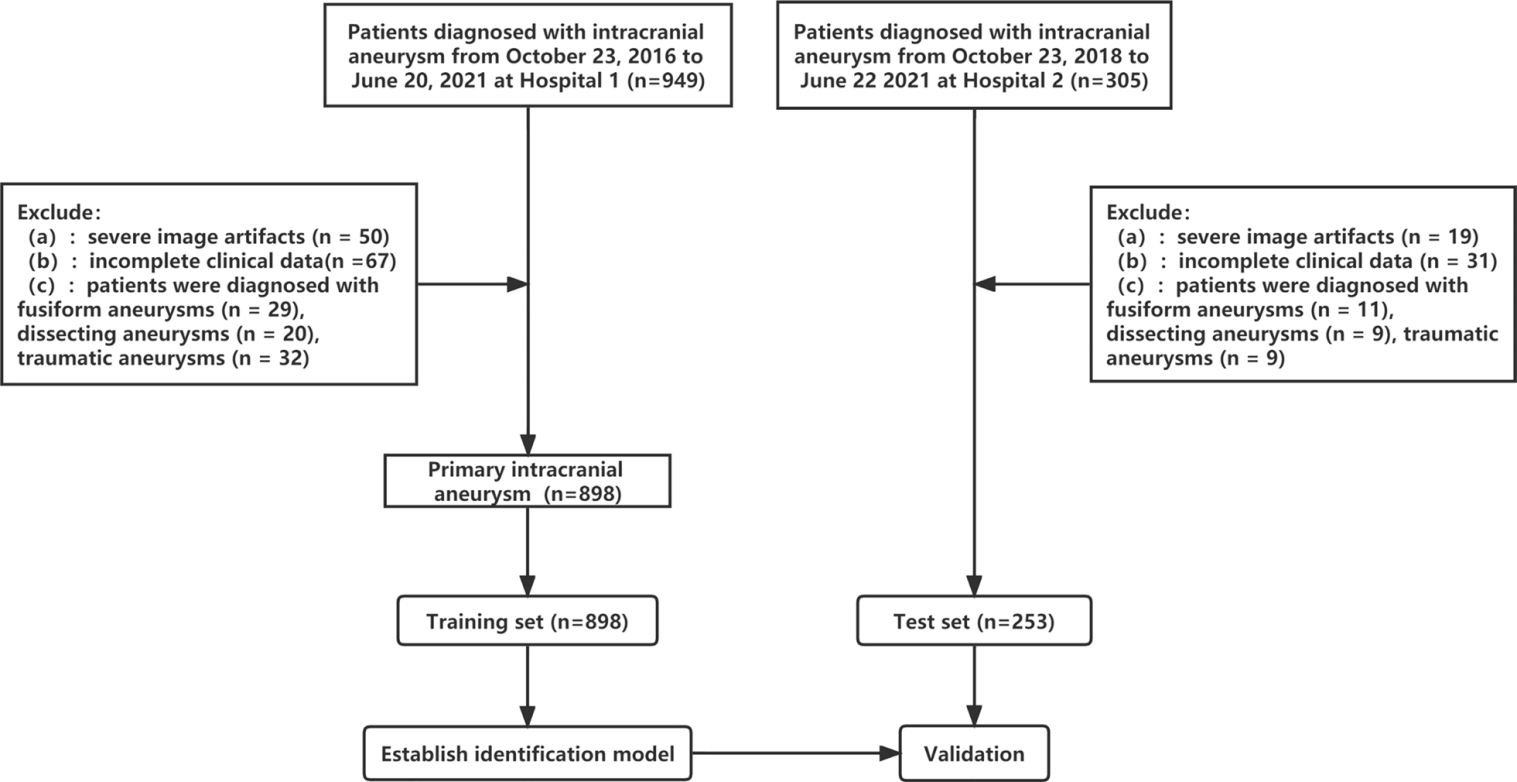
The flow chart of the study design

1007 patients diagnosed with intracranial aneurysms from October 23, 2016, to June 22, 2021, in Chongqing University Central Hospital (Hospital 1, 781 patients) and the Second Affiliated Hospital of Chongqing Medical University (Hospital 2, 226 patients) were retrospectively included. The inclusion criteria for this study were (1) adult patient over 18 years old and (2) a diagnosis of intracranial aneurysm on CT angiography by a team of two neuroradiologists with more than 8 years of experience. In case of uncertainty, DSA was used for further confirmation. Exclusion criteria included: (a) severe image artifacts; (b) incomplete clinical data; (c) fusiform, dissecting, traumatic, infected aneurysms. All aneurysms were divided into two groups: the ruptured group included aneurysms with nearby spontaneous subarachnoid hemorrhage documented by conventional brain CT or confirmed by digital subtraction angiography, without any other potential pre-disposing factor (trauma, dissection, or local or systemic infection), and the unruptured group included aneurysms with no subarachnoid hemorrhage or related clinical symptoms. 168 patients from Hospital 1 were excluded, including 29 patients with fusiform aneurysms, 20 patients with dissecting aneurysms, 2 patients with traumatic aneurysms, 50 patients with severe image artifacts and 67 patients with incomplete clinical data. 79 patients from Hospital 2 were excluded, including 11 patients with fusiform aneurysms, 9 patients with dissecting aneurysms, 9 traumatic aneurysms, 19 patients with severe image artifacts and 31 patients with incomplete clinical data. The flow chart of the study design is shown in Fig. 1.

### Clinical data collection and cerebral CTA

Clinical data included gender, age, smoking status (smoking for more than ten years), alcoholism (alcohol dependence and addiction), and hypertension. All head CTA images were collected from four different CTs: United Imaging 760 64, Shanghai, China; General Electric Company, LightSpeed 64, USA; General Electric Company, Optima 660 64, USA; Canon Medical, Canon Aquilion ONE 320, Toshiba, Japan. Similar scanning parameters were used for all scans: 100 to 120 kV and automatic mas; section thickness: 0.5 to 0.625 mm; screw pitch: 0.984 to 0.975; reconstruction thickness: 1 mm; reconstruction interval: 0.5 to 0.625 mm. Contrast agent was injected by smart tracking method. Each patient had a standard intravenous cannula placed in the anterior cubital vein in the upper limb and contrast was injected using an automatic double syringe. 50 mL iohexol (350mgI/ml) and 30 mL normal saline were intravenous bolus injected. The injection rate is 4.0 mL/s. The CTA scan was initiated using a trajectory tracking technique, where a trigger area was manually placed near the ascending aorta with a threshold of 110–120 HU and a delay of 8–10 s.

### Aneurysm segmentation and morphological feature measurement

**Fig. 2:**
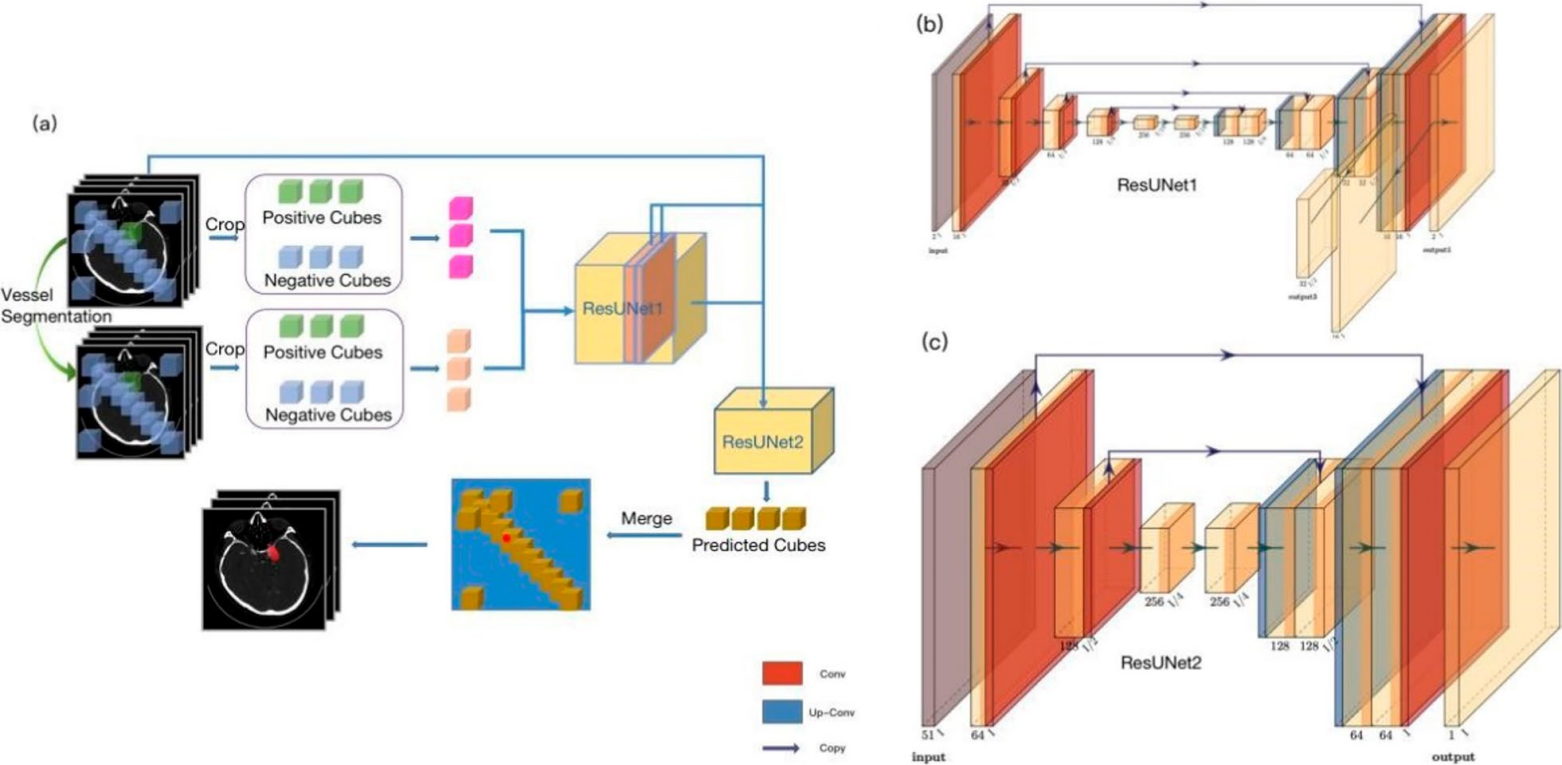
Aneurysm Segmentation network overview. **a** The complete pipeline of AS network. 3D patches were cropped uniformly from original CTA images and corresponding vessel segmentation images. Patches were balanced between positive (containing aneurysm) and negative types. Both original CTA and vessel segmentation patches were sent to ResUNet1 to firstly detect aneurysm with two channels of output, a probability map and corresponding aneurysm size map. Combined with original CTA images, two output channels, two layers of ResUNet1 were resized and input to ResUNet2 for segmentation. Emerging predicted cubes from ResUNet2 obtained the predicted whole volume. **b** ResUNet1 architecture illustration. **c** ResUNet2 architecture illustration

One 3-dimensional CNN network (Aneurysm Segmentation), which was embedded in CerebralDoc^®^, was applied to identify, segment aneurysms from CTA images and calculate the morphological features. It was comprised of two cascaded ResUNet models. ResUNet1 elaborated on aneurysm detection and ResUNet2 was responsible for segmentation. Due to great imbalanced ratio of aneurysm to whole background, vessel segmentation [17] was firstly performed to help remove noises in aneurysm detection. Data augmentations, such as rotation, scaling and flipping were carried out to diversify training samples and improve model robustness. All original CTA images and segmented vessels were patched into 128 × 128 × 128 cubes (Fig. 2). Detailed information was introduced in supplementary files.

Morphological features of target aneurysm and parent vessel were automatically calculated. Totally, 17 aneurysm features and 4 parent vessel features, including diameter, width, height, volume, neck plane diameter, intersection angle, size ratio, aspect ratio, etc., were obtained. Detailed information about each morphological feature was listed and explained in supplementary materials.

### Pyradiomics feature extraction

**Fig. 3:**
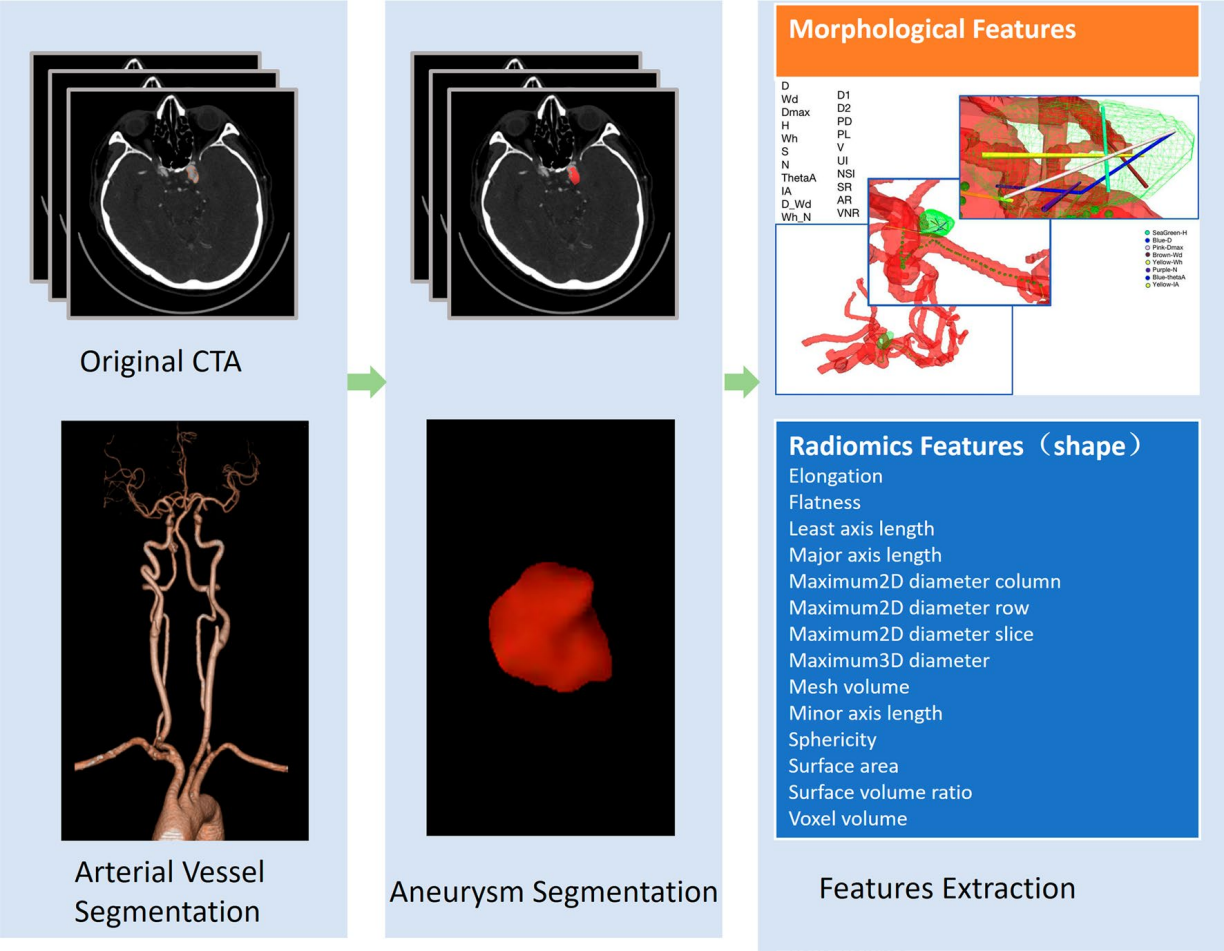
The flow chart of aneurysm identification, segmentation and feature extraction

Radiomics [18] feature extraction were performed with the open-source PyRadiomics package (version 3.0.1). Images for each patient were normalized by centering to the mean standard deviation, resampled to voxel size of 1 × 1 × 1 mm^3^ with B-Spline interpolation and grey-level discretized by a fixed bin width of 25 in the histogram. Features of elongation, flatness, least axis length, major axis length, maximum2D diameter column, maximum2D diameter row, maximum2D diameter slice, maximum3D diameter, mesh volume, minor axis length, sphericity, surface area, surface volume ratio and voxel volume were extracted [19, 20]. Detailed information of each feature was available in following website https://pyradiomics.readthedocs.io/en/latest/ or online documentation [21]. All features were subjected to Z-score normalization before feeded to models (Fig. 3).

### Dimensionality reduction and model construction

To identify the most relevant features for aneurysm rupture judgment, selectKBest and L2-based logistic regression algorithms were successively applied in training dataset after preprocessing. All features with p value less than 0.05 after selectKBest were considered as statistically significant candidates for logistic regression model. L2-based regularization was used to obtain the most relevant features. Cross-validation was carried out to establish the optimal hyparameters for l2-based logistic regression model. Support vector machine (SVM), random forest (RF) and multilayer perceptron (MLP) were used to establish models to distinguish ruptured and unruptured aneurysms. Grid search with fivefold cross-validation was applied to optimize the model hyper-parameters in training dataset and then applied in the external test dataset to comprehensively estimate model performance. Sensitivity, specificity, area under the receiver operating curve (AUC) were utilized to evaluate the model performance ability. Sensitivity and specificity of each model were determined at the optimal cutoff point on AUC, that is, the point where the Youden index (*J* = sensitivity + specificity−1) reaches its maximum. Delong test was used to compare the performance ability among RF, SVM and MLP models by sklearn package (version 0.23.2) in Python (version 3.8.12).

All statistical analyses were performed using the Statistical Package for Social Sciences software version 23.0 (SPSS Inc., Chicago, Illinois, USA). Two-sample t-test was used for continuous variables, and chi-square test was used for discrete variables. *P* values less than 0.05 were considered statistically significant.

## Results

### Patient demographics

The demographic, clinical, and radiographic characteristics are summarized in Table 1.

**Table 1:** Patients’ characteristics in the training cohort and testing cohort

Training cohort			Testing cohort		
Variables	Ruptured (*n* = 363)	Unruptured (*n* = 535)	Variables	Ruptured (*n* = 63)	Unruptured (*n* = 190)
Age	58.04 ± 12.54	66.43 ± 12.61	Age	59.05 ± 11.80	65.48 ± 11.08
Gender			Gender		
Male	172 (47.4%)	234 (56%)	Male	25 (39.7%)	83 (50.9%)
Female	191 (52.6%)	184 (44%)	Female	38 (60.3%)	80 (49.1%)
Smoking	81 (22.3%)	109 (26.1%)	Smoking	18 (28.6%)	45 (26.6%)
Alcoholism	57 (15.7%)	39 (9.3%)	Alcoholism	12 (19.0%)	28 (17.2%)
Hypertension	304 (83.7%)	304 (72.7%)	Hypertension	26 (41.3%)	93 (57.1%)
Location			Location		
ACA	117 (32.2%)	47 (8.8%)	ACA	11 (17.5%)	17 (0.9%)
ICA	171 (47.1%)	399 (74.6%)	ICA	40 (63.5%)	150 (78.9%)
MCA	58 (16.0%)	61 (11.4%)	MCA	11 (17.5%)	16 (8.4%)
PCA	17 (4.7%)	28 (5.2%)	PCA	1 (1.5%)	7 (3.7%)

363 ruptured aneurysms and 535 unruptured aneurysms from Hospital 1 were included in the training set. 63 ruptured aneurysms and 190 unruptured aneurysms from Hospital 2 were used for independent external testing. For Hospital 1, the locations of ruptured aneurysms were as follows: anterior cerebral arteries or anterior communicating arteries (117 cases, 32.2%), internal carotid arteries (171 cases, 47.1%), middle cerebral arteries (58 cases, 16%) and posterior circulation arteries (17 cases, 4.7%). 81 (22.3%) patients had smoking, 57 (15.7%) patients had alcoholism, and 304 (83.7%) patients had hypertension. The locations of unruptured aneurysms were as follows: anterior cerebral arteries or anterior communicating arteries (47 cases, 8.8%), internal carotid arteries (399 cases, 74.6%), middle cerebral artery (61 cases, 11.4%) and posterior circulation arteries (28 cases, 5.2%). 109 (26.1%) patients had smoking, 39 (9.3%) patients had alcoholism and 304 (72.7%) patients had hypertension.

Patients from Hospital 2 were all used for independent external testing. The locations of ruptured aneurysms were as follows: anterior cerebral arteries or anterior communicating arteries (11 cases, 17.5%), internal carotid arteries (40 cases, 63.5%), middle cerebral arteries (11 cases, 17.5%) and posterior circulation arteries (1 cases, 1.6%). 18 (28.6%) patients had smoking, 12 (19.0%) patients had alcoholism, and 26 (41.3%) patients had hypertension. The locations of unruptured aneurysms were as follows: anterior cerebral arteries or anterior communicating arteries (17 cases, 8.9%), internal carotid arteries (150 cases, 78.9%), middle cerebral artery (16 cases, 8.4%), posterior circulation arteries (7 cases, 3.7%). 45 (26.6%) patients had smoking, 28 (17.2%) patients had alcoholism and 93 (57.1%) patients had hypertension.

### Feature selection and model performance

**Fig. 4:**
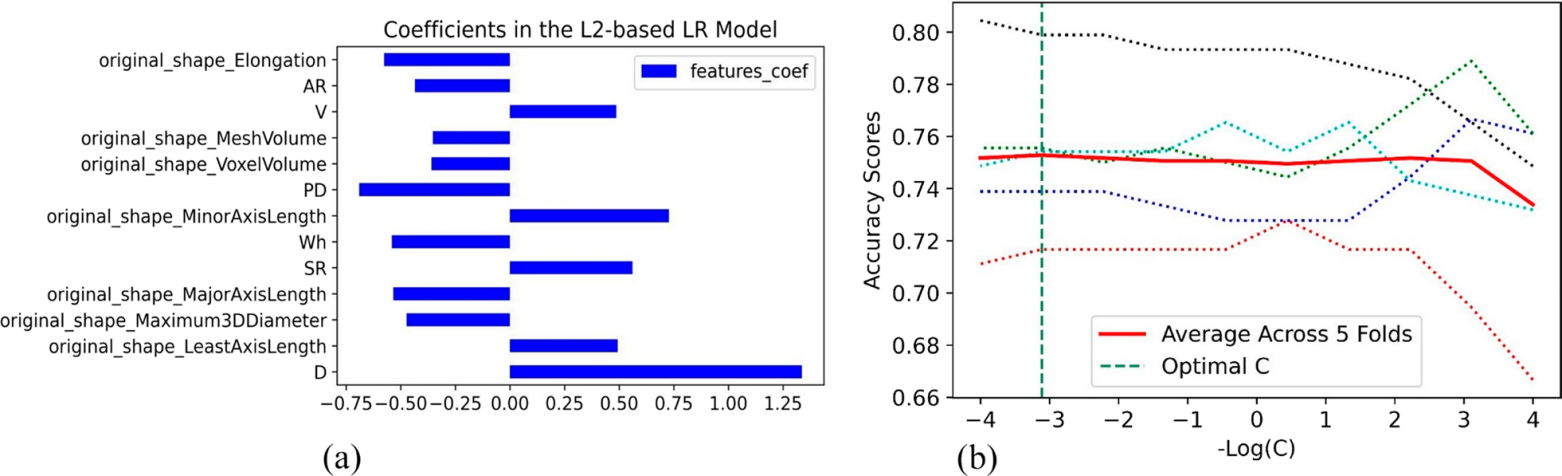
**a** The feature coefficients of L2-based LR model. **b** The optimal C was selected via five-fold cross-validation

For each aneurysm, 21 morphological features were automatically calculated by CerebralDoc^®^ and 14 radiomics features were obtained by Pyradiomics. After selectKBest, 33 features were retained. 13 features with absolute coefficients larger than the mean in L2-based logistic regression model were considered as most relevant features to determine rupture status, including 7 radiomics features, 1 parent vessel feature and 5 aneurysm features (Fig. 4A). The best C for L2-based logistic regression is 1291.55, selected via fivefold cross-validation determined by accuracy (Fig. 4B).

**Fig. 5:**
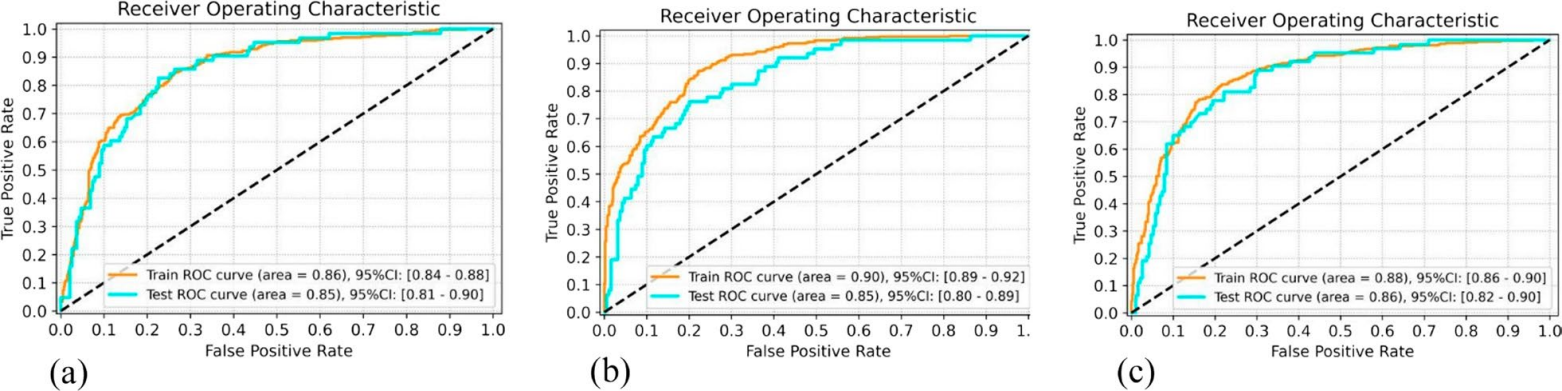
Receiver operating characteristic (ROC) curves of SVM (**a**), RF (**b**), MLP (**c**) models in the training and testing dataset

In the training set, the AUCs of SVM, RF and MLP were 0.86, 0.90, 0.88, respectively. In the external test set, the AUCs of SVM, RF and MLP were 0.85, 0.85, 0.86, respectively. Detailed sensitivity and specificity are plotted in Fig. 5. Delong tests showed that there was no significant difference among the three models (SVM versus RF, *p* = 0.76, z score = 0.30; SVM versus MLP, *p* = 0.57, z score = 0.56; RF versus MLP, *p* = 0.43, z score = 0.79).

## Discussion

Evaluating the status of intracranial aneurysm is the key to select clinical treatment strategies. Early diagnosis and treatment of ruptured aneurysms can significantly reduce the mortality and disability rate. In this study, SVM, RF and MLP models were established to discriminate ruptured and unruptured intracranial aneurysms based on CTA images. They all showed high accuracies of 0.85 to 0.86 in external validation data. Our results suggested that these methods have great potential value in clinical practice. They could enable neurosurgeons and interventional physicians to better assess aneurysm status and plan surgery. Previously, Liu et al. [22] extracted morphological features of 719 aneurysms to predict the stability of aneurysms with an AUC of 0.73. Zhu et al. [14] used radiomics features to identify ruptured and unruptured aneurysms in the middle cerebral arteries, and obtained an AUC of 0.738 in the validation set. Compared with these previous studies [22, 23], our study had the largest sample size and the highest accuracy. External verification was adopted, and better reliability was obtained. On the other hand, all previous studies used traditional manual methods to segment, reconstruct aneurysms and measure morphological parameters. It took at least 30 min for one patient, which greatly reduced the efficiency of clinical diagnosis. Manual depiction of aneurysms led to huge workload and visual fatigue of doctors, which might reduce the accuracy. In the actual clinical work, this method was not feasible for emergency patients. Our study was the first time to use artificial intelligence model to automatically detect, segment aneurysms on CTA images. Our fully automatic model could quickly process the data in 1 min to obtain real-time results with good stability. For patients with multiple aneurysms accompanied by subarachnoid hemorrhage in daily clinical work, CTA images were uploaded to the software platform, and through platform analysis criminal aneurysms could be automatically identified and labeled. With this system, doctors could quickly find criminal aneurysms and deal with them in time. This could greatly improve the medical efficiency and save medical costs.

In this study, 33 features were extracted from each aneurysm to establish the classification model for ruptured and unruptured aneurysms discrimination. After final feature selection, 13 features were retained that were considered to be associated with aneurysm rupture, including 6 morphologic features and 7 radiomics features. The six morphological features included D (diameter), V (volume), Wh (the largest distance orthogonal to height), AR (aspect ratio), SR (size ratio) and PD (parent vessel diameter). The D (diameter) was the largest distance between the center of the neck and the aneurysm surface that fitted inside the aneurysm dome. Numerous studies [24, 25] have shown that the larger the D or volume of the aneurysm, the more likely it is to rupture. The International Study of Unruptured Intracranial Aneurysms (ISUIA) [26] results have shown a 5-year rupture rate of 14.5% for 13 to 24 mm in diameter and if the diameter is greater than 25 mm, the 5-year fracture rate is 40%. Wh was the largest distance orthogonal to height. Kim et al. studied 57 patients with ruptured aneurysms and 198 patients with unruptured aneurysms, and found that vertical height was the only significant predictor of rupture. AR was defined as the ratio between D and the maximum diameter of the neck plane. Previous studies [27] have proved that larger neck diameters and AR > 1.5 were key factors for posterior communicating artery aneurysms rupture. PD was the weighted average diameter of parent vessel, starting from 5 mm close to proximal point and ending at 5 mm close to distal point of the neck plane [28]. It was the only retained parent vessel feature. SR (size ratio) was defined as the ratio between D and PD. Rahman et al. [29] had proved SR was associated with the rupture state of IA through blind test. The greater the SR, the higher the risk of aneurysm rupture. The 7 radiomics features were all shape features, including elongation ratio, mesh volume, voxel volume, major Axis length, minor axis length, least axis length and maximum 3D diameter. The elongation ratio showed the relationship between the two largest principal components in the aneurysm. Small elongation had been proved important factors of aneurysm rupture [19]. Mesh volume and voxel volume mainly reflect the size of aneurysm. Previous studies [25, 26] have found that volume change was an independent factor related to the formation of irregular intracranial aneurysms, which may directly lead to aneurysm rupture. The major axis length, minor axis length and least axis length represent the maximum axis length, the second axis length and the minimum axis length of the aneurysm, respectively. The difference of axial length may induce the hemodynamic changes of intracranial aneurysms, thus affecting their stability. Maximum 3D Diameter was defined as the largest pairwise Euclidean distance between the vertices of the tumor surface mesh. This feature could reflect the complex parameters of the aneurysm from a three-dimensional perspective, which cannot be achieved manually.

There were several limitations in our study. Firstly, this was a retrospective, cross-sectional study. A prospective and long-term follow-up study is needed to further validate our models. Secondly, all subjects included in the study were Asians. Different races or countries have different biology and etiology, which may enhance model robustness. Finally, many patients with subarachnoid hemorrhage were excluded due to immediate surgery. The imbalance between the number of ruptured and unruptured cases may affect the accuracy of our model. In future research, we will strive to overcome the above limitations, obtain multi-ethnic and multi-national samples, and design a prospective dynamic longitudinal study to verify the reliability of our research results.

## Conclusion

In this study, we established three classification models which could distinguish ruptured and unruptured aneurysms accurately. Our fully automatic model could rapidly process the CTA data and evaluate the status of aneurysms in one minute. It greatly improves the diagnostic efficiency and has important value to help the early diagnosis and treatment in clinical practice.

## Data Availability

The datasets used and/or analyzed during the current study are available from the corresponding author on reasonable request.

## Abbreviations

AR: Aspect ratio
AUC: Receiver operating curve
CNN: Convolutional neural network
CTA: Computed tomography angiography
DSA: Digital subtraction angiography
IAs: Intracranial aneurysm
ISUIA: Unruptured intracranial aneurysms
MLP: Multilayer perceptron
PD: Parent vessel diameter
RF: Random forest
SAH: Subarachnoid hemorrhage
SR: Size ratio
SV: Support vector machine
Wh: The largest distance orthogonal to height
